# Role of the renin-angiotensin system in the development of COVID-19-associated neurological manifestations

**DOI:** 10.3389/fncel.2022.977039

**Published:** 2022-09-16

**Authors:** Lucía A. Méndez-García, Galileo Escobedo, Alan Gerardo Minguer-Uribe, Rebeca Viurcos-Sanabria, José A. Aguayo-Guerrero, José Damián Carrillo-Ruiz, Helena Solleiro-Villavicencio

**Affiliations:** ^1^Laboratory of Immunometabolism, Research Division, General Hospital of Mexico “Dr. Eduardo Liceaga,” Mexico City, Mexico; ^2^Laboratory of Molecular Neuropathology, Cellular Physiology Institute, National Autonomous University of Mexico, Mexico City, Mexico; ^3^PECEM, School of Medicine, National Autonomous University of Mexico, Mexico City, Mexico; ^4^Research Directorate, General Hospital of Mexico “Dr. Eduardo Liceaga,” Mexico City, Mexico; ^5^Department of Neurology and Neurosurgery, General Hospital of Mexico “Dr. Eduardo Liceaga,” Mexico City, Mexico; ^6^Facultad de Ciencias de la Salud, Universidad Anáhuac, Huixquilucan, Mexico; ^7^Posgrado en Ciencias Genómicas, Universidad Autónoma de la Ciudad de México, Mexico City, Mexico

**Keywords:** SARS-CoV-2, renin-angiotensin system, ACE2, neuroinflammation, neurological manifestations, COVID-19, long COVID

## Abstract

SARS-CoV-2 causes COVID-19, which has claimed millions of lives. This virus can infect various cells and tissues, including the brain, for which numerous neurological symptoms have been reported, ranging from mild and non-life-threatening (e.g., headaches, anosmia, dysgeusia, and disorientation) to severe and life-threatening symptoms (e.g., meningitis, ischemic stroke, and cerebral thrombosis). The cellular receptor for SARS-CoV-2 is angiotensin-converting enzyme 2 (ACE2), an enzyme that belongs to the renin-angiotensin system (RAS). RAS is an endocrine system that has been classically associated with regulating blood pressure and fluid and electrolyte balance; however, it is also involved in promoting inflammation, proliferation, fibrogenesis, and lipogenesis. Two pathways constitute the RAS with counter-balancing effects, which is the key to its regulation. The first axis (classical) is composed of angiotensin-converting enzyme (ACE), angiotensin (Ang) II, and angiotensin type 1 receptor (AT1R) as the main effector, which -when activated- increases the production of aldosterone and antidiuretic hormone, sympathetic nervous system tone, blood pressure, vasoconstriction, fibrosis, inflammation, and reactive oxygen species (ROS) production. Both systemic and local classical RAS’ within the brain are associated with cognitive impairment, cell death, and inflammation. The second axis (non-classical or alternative) includes ACE2, which converts Ang II to Ang-(1–7), a peptide molecule that activates Mas receptor (MasR) in charge of opposing Ang II/AT1R actions. Thus, the alternative RAS axis enhances cognition, synaptic remodeling, cell survival, cell signal transmission, and antioxidant/anti-inflammatory mechanisms in the brain. In a physiological state, both RAS axes remain balanced. However, some factors can dysregulate systemic and local RAS arms. The binding of SARS-CoV-2 to ACE2 causes the internalization and degradation of this enzyme, reducing its activity, and disrupting the balance of systemic and local RAS, which partially explain the appearance of some of the neurological symptoms associated with COVID-19. Therefore, this review aims to analyze the role of RAS in the development of the neurological effects due to SARS-CoV-2 infection. Moreover, we will discuss the RAS-molecular targets that could be used for therapeutic purposes to treat the short and long-term neurological COVID-19-related sequelae.

## Introduction

The severe acute respiratory syndrome coronavirus 2 (SARS-CoV-2) is the causal agent of coronavirus disease 2019 (COVID-19), which mainly exhibits respiratory and cardiovascular complications and a range of neurological manifestations ranging from mild and non-life-threatening (e.g., headaches, anosmia, dysgeusia, and disorientation), to severe and life-threatening symptoms (e.g., meningitis, ischemic stroke, and cerebral thrombosis). Studies show that up to 80% of patients hospitalized with COVID-19 present neurological complications during and even after the infection is overcome (Bauer et al., 2022). Potentially these complications are -partially- due to an imbalance in the renin-angiotensin system (RAS) since, to infect the host cells, SARS-CoV-2 binds to angiotensin-converting enzyme 2 (ACE2), an enzyme belonging to non-classical RAS (Harapan and Yoo, 2021).

RAS is one of the most complex hormonal systems regulating blood pressure, fluid, and electrolyte balance. However, it has recently been associated with multiple cellular mechanisms, including inflammation, proliferation, lipogenesis, and fibrogenesis. RAS consists of two regulatory pathways, the classical and non-classical, alternative, or protector pathway, which have opposing effects, being this the key to its function. Depending on the site of action, RAS can be either peripheral or local. Each of these systems has components that make them unique and play a critical role in health and disease.

In COVID-19, the binding of SARS-CoV-2 to ACE2 reduces this enzyme’s activity and disrupts the balance of RAS, therefore, the accumulation of Ang II leads to harmful effects. In that sense, the binding of Ang II to AT1R in the brain promotes the activation of pro-inflammatory and pro-oxidant signaling pathways leading to endothelial dysfunction, thrombo-inflammatory processes, and reduction of cerebral irrigation, resulting in cerebrovascular complications (Tsivgoulis et al., 2020), which could partially explain the appearance of some of the neurological symptoms associated with COVID-19. Thereby, this review aims to analyze the role of RAS in the development of the neurological manifestations due to SARS-CoV-2 infection, and will discuss the RAS-molecular targets that could be used for therapeutic purposes to treat the short and long-term neurological COVID-19-related sequelae with particular emphasis on angiotensin receptor blockers (ARBs).

## Renin-angiotensin system

Studies related to systemic RAS began in 1898 when renin was identified as a substance that increases blood pressure; however, it took about 50 years to identify all components of the classical RAS (Paul et al., 2006).

Two pathways constitute RAS. The classical axis is composed of ACE, Ang II, and AT1R as the main effector that mediates the biological actions of Ang II (Campbell, 2014; Vargas Vargas et al., 2022). Renin, the first enzyme of RAS, is produced in the juxtaglomerular cells of the afferent renal arteriole in response to glomerular hypoperfusion. Circulating renin cleaves its substrate, angiotensinogen, to form the decapeptide Ang I, which is converted to Ang II by ACE (Campbell, 2014). The classical RAS axis contributes to pathophysiological changes, including excessive renal sodium reabsorption, abnormal vascular smooth muscle cell contraction, inappropriate cardiovascular responses, and excessive aldosterone secretion (Silva et al., 2017). The aldosterone can stimulate and recruit immunological cells such as monocytes/macrophages and dendritic cells, favoring an inflammatory state and a T-cell response.

The non-classical axis comprises ACE2, which acts mainly on Ang II and converts it into Ang-(1-7). ACE2 converts Ang-(1-7) from Ang II or can metabolize Ang I into Ang-(1-9); subsequently, Ang-(1-9) can then be transformed into Ang-(1-7) by the action of ACE. In parallel, the endopeptidases neprilysin, prolyl endopeptidase, and thimet oligopeptidase form Ang-(1-7) from Ang I (Chappell et al., 2014; Chappell, 2016; Rukavina Mikusic et al., 2021). Ang-(1-7) activates MasR, causing vasodilator, antiproliferative, antifibrotic, and anti-inflammatory effects (Campbell, 2014). Moreover, ACE2 hydrolyses non-RAS-derived plasma-borne and tissue-derived peptides, for instance: apelin-36, apelin-17, apelin-13, [Pyr^1^]apelin-13, kinetensin (1-9), dynorphin A 1-13, des-Arg^9^-bradykinin and neurotensin to apelin (1-35), apelin (1-16), apelin (1-12), [Pyr^1^] apelin (1-12), kinetensin (1-8), dynorphin A (1-12), des-Arg^9^-bradykinin (1-8) and [Pyr^1^] neurotensin (1-7) (Zhang et al., 2021). The apelin family comprises short-life peptides with anti-senescence, antithrombotic, and angiogenesis homeostasis properties. Moreover, peptides such as des-Arg^9^-bradykinin, neurotensin, dynorphin, and kinetensin are involved in inflammatory response (Zhang et al., 2021).

More than three decades ago, researchers began to describe a specific RAS in many tissue types denominated tissue RAS (tRAS) or local RAS, whose imbalance is involved in the progression of various human pathologies (Saravi et al., 2021). It has been demonstrated that specific organs and tissues present a tRAS, such as kidneys, lungs, adipose tissue, liver, and brain (Paul et al., 2006). Thus, local RAS has been defined as “tissue-based mechanisms of Ang peptide formation that operate separately from the circulating RAS” (Campbell, 2014). Hence, local RAS can operate in an autocrine, paracrine, and intracrine manner and exhibit multiple physiological effects at cellular and tissue levels.

In addition to hemodynamic actions, local RAS have multiple functions, including regulating cell growth, differentiation, proliferation, apoptosis, generating reactive oxygen species (ROS), tissue inflammation and fibrosis, and hormone secretion. In that sense, the local RAS have alternative pathways regulated by biologically active peptides (e.g., Ang IV, Ang A, alamandine, and angioprotectin), additional specific receptors (e.g., pro-renin receptor), and alternative pathways for the generation of Ang II (e.g., renin-independent mechanisms of Ang peptide generation from Ang- (1-12) (Campbell, 2014). Furthermore, evidence suggests that local RAS may operate independently of the circulating RAS and play a pathogenic or protective role.

Furthermore, it is essential to mention that both local and peripheral RAS show sex-related differences. This may partly stem from a differential balance in the classical and alternative axes of the RAS. In males, the ACE/AngII/AT1R pathways are enhanced due to testosterone influence, whereas, in females, the balance is shifted toward the ACE2/Ang-(1-7)/MasR and AT2R pathways by the influence of 17β-estradiol and/or progesterone (Hilliard et al., 2013). Evidence suggests that “premenopausal women, as compared to aged-matched men, are protected from renal and cardiovascular disease, and this differential balance of the RAS between the sexes likely contributes. On the other hand, *ACE2* is located on the X chromosome in regions that usually escape the X chromosome inactivation; consequently, XX cells may over-express the gene encoding ACE2” (Gagliardi et al., 2020).

This review will further describe the brain’s local RAS to analyze its association with the COVID-19-associated neurological manifestations.

## Local brain renin-angiotensin system

**FIGURE 1:**
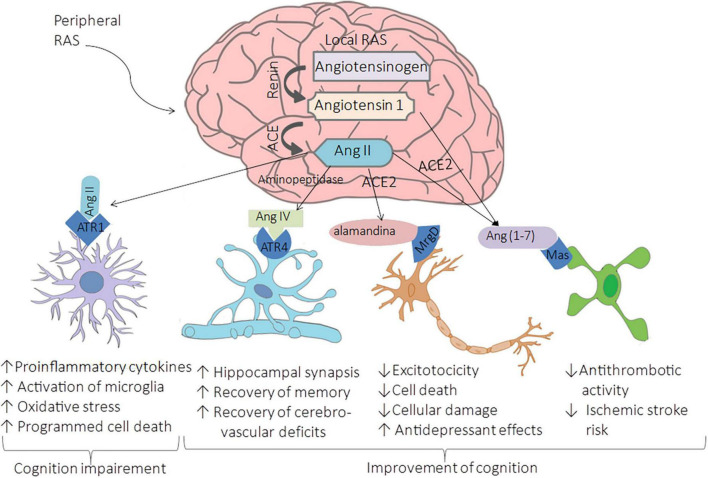
Renin-Angiotensin-System in the brain. The cleavage of cerebral angiotensinogen produces four main neuroactive peptides: Ang II, Ang IV, Ang-(1-7), and alamandine. The upregulation of ACE leads to the hyperactivation of AT1R by Ang II conducing to brain vasoconstriction associated with cognitive impairment, cell death, and inflammation. In contrast, the binding of Ang II to AT2R, the bind of Ang IV, Ang-(1-7), and alamandine to AT4R, MasR, and MrgDR respectively, are related to enhancing cognition, synaptic remodeling, cell survival, cell signal transmission, and antioxidant/anti-inflammatory mechanisms leading to improvement of cognition.

The brain has both peripheral and local RAS. Peripheral RAS from the forebrain pathway consists of capillaries that allow the access of RAS components to the brain at circumventricular organs, which lacks the blood-brain barrier (BBB) (Fry and Ferguson, 2007). The evidence of local brain RAS (brain RAS) is the expression of RAS components such as angiotensin-converting enzyme (ACE), angiotensin type 1 receptor (AT1R), AT2R, and MasR in neurons and glia. Furthermore, brain RAS seems to be mediated by the third level of regulation since the neurons express their own organelle-specific RAS components (intracrine RAS or iRAS) (Labandeira-Garcia et al., 2021), and the brain has an exclusive isoform of renin named b-renin (Shinohara et al., 2016). Besides that, it has been demonstrated that the levels of renin are low in the brain; however, there are higher levels of secreted precursor form that provides catalytic properties like those of renin named prorenin, which can bind to prorenin receptors (PRRs) that are abundant within the brain (Grobe et al., 2008). Thus, local synthesis of RAS is essential, and astrocytes play a pivotal role in producing almost 90% of the brain’s angiotensinogen, secreted for conversion into Ang and then cleaved into various neuroactive peptides (Bodiga and Bodiga, 2013). There are four main neuroactive angiotensin-derived peptides within the brain: Ang II, Ang IV, Ang-(1-7), and alamandine (Jackson et al., 2018), as shown in Figure 1.

As in systemic RAS, in the brain RAS, Ang II results from angiotensinogen cleavage by renin to Ang I, then processed by ACE. This neuroactive peptide binds to AT1R, located on neurons, oligodendrocytes, microglia of the cortex, and astrocytes. The upregulation of ACE leads to the hyperactivation of AT1R by Ang II, conducting to brain vasoconstriction associated with cognitive impairment, cell death, and inflammation (Labandeira-Garcia et al., 2017). AT1R promotes inflammation by the secretion of proinflammatory cytokines and the activation of microglia; this activation also produces oxidative stress that activates programmed cell death in specific regions with cognition functions such as the cortex and hippocampus, conducing to cognition impairment (Wu et al., 2022; Figure 1). These effects are due to the increment in the ROS by the induction of NADPH oxidase 2 (NOX2) and NOX4 axes (Peng et al., 2013). Contrary to the binding of Ang II to AT1R, the binding of Ang IV, Ang-(1-7), and alamandine to their respective receptors are related to enhancing cognition, synaptic remodeling, cell survival, cell survival, and cell signal transmission, and antioxidant/anti-inflammatory mechanisms (Figure 1).

Ang IV is a product of the processing of Ang II by consecutive actions of aminopeptidase A and N to generate Ang III and subsequently Ang IV (Jenkins et al., 2007). This neuroactive peptide binds to angiotensin type 4 receptor (AT4R) and when in high concentrations, binds to AT1R (Wang et al., 2018). AT4R is restricted to neurons, an insulin-regulated aminopeptidase located in the basal ganglia, cortex, and hippocampus (Abrahão et al., 2019). Studies performed in animal models demonstrated that the Ang IV interacts with the brain hepatocyte growth factor/c-Met receptor system leading to improved cognition and increased hippocampal synaptic connectivity (Wright et al., 2015). Moreover, the intervention of Ang IV in a mouse model of Alzheimer’s Disease (AD) contributes to the recovery of memory and cerebrovascular deficits (Royea et al., 2020).

ACE2 is a critical enzyme in the role of vasodilation, cognition improvement, antiproliferative, antioxidant, and anti-inflammatory effects within the brain producing Ang-(1-7) and alamandine [also known as (Ala1)-Ang-(1-7)] that bind to MasR and Mas-related G-protein coupled receptor of the type D (MrgD), respectively. Evidence suggests that the Ang-(1-7)/MasR axis shows favorable effects on cerebrovascular disease in animal models. For instance, the interaction between Ang-(1-7) and nitric oxide or bradykinin provides an antithrombotic activity that reduces the risk of ischemic stroke, mainly in older animals (Zheng et al., 2014). On the other hand, alamandine has a high affinity to MrgD; it is a neuroactive peptide formed by the decarboxylation of Ang-(1-7) and alternatively by the cleavage of angiotensin A (also known as [Ala1]-Ang II obtained by decarboxylation of aspartate to alanine on Ang II molecule) by ACE2 (Cosarderelioglu et al., 2020). The intervention of alamandine in *in vitro* and *in vivo* brain ischemia models protects from cellular damage, excitotoxicity, and cell death, showing its neuroprotective role (Gonçalves et al., 2022). Furthermore, in transgenic rats TGR (ASrAOGEN)680 with low brain angiotensinogen, the administration of alamandine induced an antidepressant-like effect measured by the immobility time in the forced swim test (Almeida-Santos et al., 2017). Moreover, cortex and hippocampal expression of Ang (1–7), MasR, and neuronal NO synthase is controlled by female sex hormones (Cheng et al., 2015), which may explain why premenopausal women have a reduced risk of cardiovascular disease and stroke when compared to men (Nakagawa and Sigmund, 2017).

All the angiotensin receptors are expressed in neurons; however, it has been a matter of debate whether ACE2 is expressed in all types of neurons or not (Brann et al., 2020). Furthermore, neurons have their own intracellular or iRAS; at mitochondrial, endoplasmic reticulum, and nuclear levels. Neurons express RAS components such as AT1R, AT2R, and MasR. Interestingly, renin-b is an intracellular isoform of renin expressed only in the brain proposing the possibility of a third level of RAS exclusively for this organ (Shinohara et al., 2016). However, the deleterious or beneficial effects of iRAS and renin-b are unclear (Labandeira-Garcia et al., 2021).

Although it has been barely explored, cerebrospinal fluid (CSF) has a particular profile of RAS. For instance, there is experimental evidence that angiotensinogen expression in the brain may be regulated independently of the peripheral angiotensinogen (produced by the liver), as it has been observed that chronic blockade of ACE decreased angiotensinogen levels in plasma but not in the CSF of spontaneously hypertensive and Wistar Kyoto rats (Schelling et al., 1983). Furthermore, *in vitro*, and *in vivo* measurements of CSF RAS components have revealed that Ang I is not generated in the CSF as renin appears unmeasurable within CSF. Accordingly, in this study, Ang I concentrations were low in CSF, while Ang II levels were comparable to those measured in plasma under control conditions (Schelling et al., 1980). In contrast, angiotensinogen and ACE were high. Furthermore, a recent study conducted by Kehoe et al. (2019) has revealed that an imbalance in the CSF RAS components is associated with disease pathology in AD. This observational study indicates that CSF ACE activity is elevated in AD and positively correlated with CSF ACE2 (Kehoe et al., 2019).

Interestingly, CSF ACE2 activity was positively associated with normal aging. Moreover, authors found in AD patients moderately strong associations between CSF ACE activity and platelet-derived growth factor beta receptor (sPDGFR), a marker of pericyte damage, and between CSF ACE2 activity and CSF albumin levels, a marker of BBB leakiness (Kehoe et al., 2019). On the other hand, in a study conducted by Xu et al., it was observed that ACE2 is the main enzyme converting Ang II into Ang-(1-7) in human CSF; also, the authors found that ACE2 is elevated in the CSF of hypertensive patients and that its levels correlated with systolic pressure (Xu et al., 2017).

Although there are few studies on the RAS components in the CSF, the evidence suggests that this fluid has specific characteristics that must be regulated since an -acute or chronic- imbalance of its components favors the classic RAS axis, which is associated with impairment of the BBB, which can lead to the development of neurological diseases. In this sense, specific components of RAS could serve as biomarkers in the CSF that help diagnose and treat neurological disorders associated with COVID-19.

In addition, the complexity of the RAS in the brain increases because, even when the ACE2 expression has been reported in several organs, including the human brain, the cell-specific expression pattern is still unknown. According to transcriptome meta-analysis, ACE2 is expressed in several brain regions, from the stratium (choroid plexus to paraventricular nuclei of the thalamus) to the cortex (the middle temporal gyrus and posterior cingulate cortex) (Kabbani and Olds, 2020). Recent studies show that the higher expression of ACE2 is in the pons and medulla oblongata in the human brainstem (Lukiw et al., 2022). In contrast, *in vitro* studies using human CD31^+^ brain endothelial cells show that the expression of ACE2 is below the threshold of detection; however, the expression increases under fluid shear stress in the 3D model system, and this increment favors the SARS-CoV-2 infection through spike protein. Therefore, the ACE2 expression could be variable due to the differences in an arterial or venous steady flow in the brain in a specific manner determining the individual risk for neurological complications during or after the infection by SARS-CoV-2 (Kaneko et al., 2021).

## SARS-CoV-2 and COVID-19

SARS-CoV-2 is a beta coronavirus belonging to the same subgenus as the SARS-CoV and the Middle East Respiratory Syndrome Coronavirus (MERS-CoV) (Cascella et al., 2022). SARS-CoV-2 is an enveloped, non-segmented, positive-sense, single-stranded RNA virus with a genome of 30 kilobases that encodes 7,096 aminoacids that constitute four structural proteins [spike (S), envelope (E), membrane (M) and nucleocapsid (N)], and 15 non-structural proteins (NSP1, NSP2, NSP3, NSP4, NSP5, NSP6, NSP7, NSP8, NSP9, NSP10, NSP12, NSP13, NSP14, NSP15, and NSP16), and eight accessory proteins (3a, 3b, 6, 7a, 7b, 8b, 9b, and ORF14) (Lamptey et al., 2021; da Silva Torres et al., 2022).

SARS-CoV-2 uses the same receptor as SARS-CoV, ACE2; however, the affinity of SARS-CoV-2 for ACE2 is about 20 times higher than that of SARS-CoV, which partially explains its higher transmissibility (Huo et al., 2020). The entry route for SARS-CoV-2 into host cells is through the Spike (S) protein, which binds to ACE2 on host cells. The S protein is a homotrimer composed of monomers of an N-terminal S1 subunit (responsible for binding to the receptor) and a C-terminal S2 subunit (responsible for fusion with the host cell membrane). On the other hand, the receptor-binding domain (RBD) of protein S directly interacts with ACE2 (Huo et al., 2020). The S protein is initially prefused where the S1 domains cover the top of S with the RBD located at the tip. The RBD is predominantly in an “inactive” state. The receptor-binding site is inaccessible; however, it undergoes conformational changes that cause it to rotate with a hinge-like motion, thus transiently presenting the ACE2 receptor-binding site (Huo et al., 2020). When ACE2 is blocked, it keeps RBD “up,” so the S1 layer becomes destabilized. This possibly favors conversion to a post-fusion form, in which S2, through massive conformational changes, drives its domain from upward fusion to dock with the host membrane (Huo et al., 2020). After fusion occurs, type II transmembrane serine protease (TMPRSS2), present on the host cell surface, removes ACE2 and activates receptor-bound S proteins; this will cause conformational changes that allow the virus to enter cells (Astuti and Ysrafil, 2020). After entering cells, SARS-CoV-2 releases its genomic material into the cytoplasm, where the viral replication process will begin (Astuti and Ysrafil, 2020; Pastrian-Soto, 2020).

The incubation period of COVID-19 goes from 5 to 6 days but can be up to 14 days. During this period (“pre-symptomatic” period), the infected individuals can be contagious and transmit the virus to healthy individuals in the population. After this time frame, many COVID-19 patients manifest a range of signs and symptoms such as fever, body aches, breathlessness, malaise, and dry cough; on the other hand, patients may present with asymptomatic, mild, moderate, or severe disease (Parasher, 2021). Furthermore, some patients also experiment neurological complications, which include confusion, stroke, and neuromuscular disorders, that generally appear during acute COVID-19. However, more severe symptoms such as impaired concentration, headache, sensory disturbances, depression, and even psychosis may persist for months after infection, causing “long COVID” neuropsychiatric syndromes (Zubair et al., 2020). The pathophysiological mechanisms are poorly understood, although evidence implicates immune dysfunction, including non-specific neuroinflammation and antineuronal autoimmune dysregulation. Also, dysregulation of systemic and brain RAS has been pointed out as a possible cause of the neurological COVID-19 manifestations.

Interestingly, it has been reported that there are some sex- and gender-related differences linked to SARS-CoV-2 infection (Prinelli et al., 2022). Male patients frequently have severe COVID-19 symptoms such as fever and pneumonia, leading to worse disease progression. However, females are more likely to present atypical symptoms characterized by sore throat/rhinorrhea, gastrointestinal disturbances, headache, conjunctivitis, and palpitations that are associated with less severe outcomes (Prinelli et al., 2022). These sex differences rely on the disparities in immune responses throughout life, which are influenced by age and reproductive status. It seems that females are less susceptible to infections than their male counterparts. Female hormones (estrogens and progesterone) suppress the production of proinflammatory cytokines, which are associated with the COVID-19 cytokine storm.

Furthermore, high estrogen concentrations in premenopausal women might lead to an overexpression of ACE2 in females, protecting them by inducing anti-inflammatory responses. On the contrary, low estrogen levels in males may contribute to higher disease susceptibility and death rates (Prinelli et al., 2022). “Androgens could also promote the transcription of the TMPRSS2 gene facilitating viral entry into the cells and decreasing the antibody response to viral infections” (Prinelli et al., 2022).

## Mechanisms of SARS-CoV-2 SNC infection

SARS-CoV-2 mainly affects the respiratory system (Chen et al., 2020). Nevertheless, increasing evidence confirms that it also invades the central nervous system (CNS), detecting neurological involvement among several cases of SARS-CoV-2, with an incidence of 36.4% (Mao et al., 2020). Although neurological manifestations are primarily seen in severe cases of COVID-19, it was shown that CNS involvement in SARS-CoV-2 infection could be observed in non-severe patients because a CNS invasion can occur in both the initial and late phases of SARS-CoV-2 disease (Baig et al., 2020).

Several studies suggest that the neurological symptoms result from the virus outside the CNS because of the inflammatory mediators that this infection triggers. However, new evidence confirms that the SARS-CoV-2 virus may also enter the brain through different pathways (Zhou et al., 2020).

**FIGURE 2:**
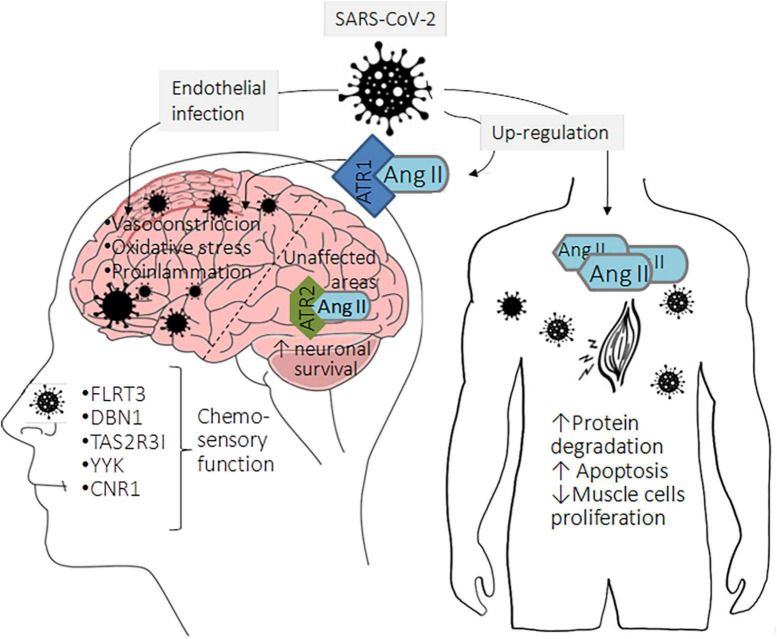
Neurological symptoms as consequence of classical RAS upregulation in COVID-19 patients. The lack of Ang II processing by ACE2 and its subsequent binding to AT1R can cause a local pro-inflammatory state that activates neuronal programmed cell death and the consequent dopaminergic neuronal loss leading to cognitive problems. Accumulating evidence indicates that brain areas unaffected by the virus show a low AT1R expression accompanied by high AT2R expression. Then, Ang II can bind to AT2R with high affinity, triggering protective mechanisms such as neuronal survival. Although previous information suggested that peripheral sensory neurons did not express ACE2, recent evidence indicates that these cells express low ACE2 levels, which may favor viral entry and infection. Subsequently, the infection leads to altered expression of genes associated with chemosensory functions, such as the olfactory neuronal markers, fibronectin leucine-rich transmembrane protein (FLRT3), drebrin 1 (DBN1), taste 2 receptor member 31 (TAS2R31), chemosensory regulatory factors Yin Yang 1 (YY1), and cannabinoid receptor 1 (CNR1). Moreover, the musculoskeletal symptoms in COVID-19 patients could be related to Ang II upregulation that orchestrates protein degradation and skeletal muscle cell apoptosis, possibly leading to loss of skeletal muscle fibers.

The CNS infection by SARS-CoV-2 can be direct or indirect through several routes: (1) retrograde axonal transport and *trans*-synaptic viral spreading, (2) *via* hematogenous considering the leakage of the BBB, (3) by the brain lymphatic drainage system (“Trojan horse” mechanism), and 4) through circumventricular organs that lack the BBB. In the following sections, we will briefly describe these mechanisms (Figure 2).

### Retrograde axonal transport and *trans*-synaptic pathway

Some viruses (e.g., adenovirus and α-herpes viruses) can migrate by infecting sensory or motor nerve endings, achieving retrograde or anterograde neuronal transport with the support of motor proteins (e.g., dynein and kinesins), which move along microtubules. Therefore, it has been proposed that SARS-CoV-2 could also have these transportation mechanisms to invade the CNS (Bodro et al., 2021). Since the olfactory nerve communicates the nasal epithelium and the olfactory bulb, viruses may enter the CNS this way (Swanson and McGavern, 2015; Meinhardt et al., 2020). Experimental data have demonstrated that coronaviruses can anterogradely reach the nervous system by crossing the neural-mucosal interface through the cribriform plate of the ethmoid bone (Rhea et al., 2020). In this context, SARS-CoV-2 can spread from the olfactory epithelium along the olfactory nerve to the olfactory bulb *via* the transcribral route, where it reaches the brain and might infect adjacent structures (Rhea et al., 2020). Retrogradely, the droplets containing SARS-CoV-2 particles that get in touch with nasal mucosa are moved along the microtubules back to neuronal cell bodies through *trans*-synaptic transport or *via* the fast axonal transport mechanism of vesicle transport (Zubair et al., 2020). Once the virus has crossed through the cribriform plate to reach the brain parenchyma, it might continue being transported to many other areas, such as the olfactory cortex of the temporal lobe to the hippocampus, and the amygdala or hypothalamus (Cheng et al., 2020). It can also penetrate the medulla oblongata’s cardiovascular control center (Meinhardt et al., 2020). The axonal transport and neuronal *trans*-synaptic propagation could be from olfactory, gustatory, trigeminal, and vagal nerves, allowing the virus to infect the brainstem in the early stages of infection (Pacheco-Herrero et al., 2021). In this pathway, primary sensory neurons communicate with the mitral cells, which have projections toward the ventricle and the medulla, leading to the spread of the virus from the CSF into the lymphatic system within the CNS and toward the peripheral nervous system (Dey et al., 2021). The trigeminal nerve branch could play a critical role, as it innervates the nociceptive cells in the nasal cavity and sensory fibers in the conjunctiva. Furthermore, the trigeminal nerve branch can carry out drug transportation, thus demonstrating the ability to endocyte, move, and exocyte proteins from a nerve ending, using the motor proteins kinesin and dynein in a retrograde way to reach the CNS (Lochhead et al., 2019).

### Hematogenous pathway

Hematogenous spread occurs because of the BBB leakage, which favors the entrance of viral particles into the CNS. The BBB integrity is primarily composed of junctional complexes containing tight adherents and gap junctions, which, when damaged, cause an increase of the permeability mainly through paracellular *via* (Luissint et al., 2012). The mechanisms of BBB disruption are diverse. Regarding SARS-CoV-2 infection, hypoxia, ischemia, and oxidative stress are common mechanisms associated with active disease (Rojas et al., 2020), which are reported to easily affect the intercellular junctions, leading to the disruption of the BBB (Sántha et al., 2016; Lee et al., 2018; Halder and Milner, 2020). At the same time, some cytokines are characterized to increase paracellular permeability *per se* (Pan et al., 2007). Blood-borne cytokines can directly cross the BBB to interact with CNS tissue (Hsuchou et al., 2012; Erickson et al., 2020). In this context, cytokines and acute phase reactants produced during the evolution of SARS-CoV-2 infection, such as IL-1β, IL-6, TNF-α, IL-17, IFN-γ, and acute-phase protein CRP have been experimentally proven to increase the BBB permeability (Hsuchou et al., 2012; Erickson et al., 2020).

Furthermore, the entry of pro-inflammatory cytokines in the brain can lead to microglial activation and proliferation that can fragment endothelial tight junction protein, further disrupting BBB’s integrity (John et al., 2003; Shigemoto-Mogami and Hoshikawa, 2018). The disruption of the BBB could, then, create tissue inflammation and release more pro-inflammatory cytokines. Moreover, resident nervous system cells, such as microglia and astrocytes, release these pro-inflammatory cytokines (Van Wagoner et al., 1999; Libbey and Fujinami, 2011; Welser-Alves and Milner, 2013). Thus, creating a self-perpetuation neuro-inflammatory milieu (Dantzer, 2018).

Moreover, it has been proposed that SARS-CoV-2 can attack *via* ACE2 receptors in the endothelial cells of blood vessels in the brain, disrupting the BBB and increasing the BBB permeability (Keyhanian et al., 2021). This mechanism is supported by the fact that viral-like particles of SARS-COV-2 have been found in Post-mortem brain endothelial capillary pericytes of COVID-19 patients (Bocci et al., 2021).

Additionally, to the BBB, SARS-CoV-2 can infect the CNS by disrupting the CSF barrier (BCSFB). The CSF constantly exchanges fluids with the blood and the interstitial fluid in a pulsatile movement throughout the brain (Brinker et al., 2014). Moreover, the BCSFB cells could activate the expression of pro-inflammatory cytokines leading to BCSFB permeability that provokes the free transit of immune cells that potentially are infected, as occurs in the mentioned “Trojan horse” mechanism, which will be reviewed below.

### Brain lymphatic drainage system (“Trojan horse mechanism”)

It has been confirmed that when SARS-CoV-2 is in an adequate milieu, it can live in human macrophages and dendritic cells from the periphery of germinal lymph nodes and peripheral blood (Yang et al., 2020; Meidaninikjeh et al., 2021; Zheng et al., 2021). *Post-mortem* examination of airway tissue confirmed that viral particles and antigens of SARS-CoV-2 are present in alveolar macrophages (Junqueira et al., 2021). Through the lymphatic drainage system, monocytes and macrophages become a viral pool that might diffuse the virus toward the CNS (Desforges et al., 2007). In the case of the macrophages, it is well known that the virus might convert these cells into long-living macrophages and promote their migration to become a viral reservoir and a vector to successfully transport the virus to hard-reachable places, including the brain (Nikitina et al., 2018; Badierah et al., 2020).

### *Via* circumventricular organs that lack the BBB

A potential way of entry of SARS-CoV-2 into the CNS is by binding to ACE2, which has been reported to be expressed on various brain cells and cerebral parts such as the subfornical organ, paraventricular nucleus, nucleus of the *tractus solitarius*, and rostral ventrolateral medulla, as well as in non-cardiovascular areas such as the motor cortex and raphe (Jakhmola et al., 2020). Some of these areas lack BBB, especially those close to the third and fourth ventricles that comprise circumventricular organs, hypothalamus, and brainstem, constantly exchanging fluids with the blood and the CSF (Quarleri and Delpino, 2022).

## Systemic renin-angiotensin system and COVID-19-associated neurological manifestations

The effects of the RAS dysregulation surpass the renal and cardiovascular systems to encompass other body tissues and organs, including the brain (Noureddine et al., 2020). Thus, it has been reported that SARS-CoV-2-infected patients can have a significant functional impairment of RAS (Osman et al., 2021; Wang et al., 2022a), which is a consequence of the binding of SARS-CoV-2’ RBD of the viral S protein, precisely, the S1 subunit to the ACE2 enzyme in human cells. As a result of this interaction, ACE2 is shedded by A disintegrin and metalloprotease 17 (ADAM17), releasing it into circulation as the soluble form of ACE2 (sACE2) (Gonzalez et al., 2021). In that sense, it has been observed that sACE2 levels increase for at least 14 days in patients with COVID-19 infection, particularly in critically ill subjects, and are higher in males than in females (Fagyas et al., 2022). However, it is essential to mention that most of these were hypertensive patients, which could represent a significant bias. Moreover, Wang et al. reported that the levels of sACE2 significantly “decreased in survivors but increased in patients who died due to COVID-19” (Wang et al., 2022). On the other hand, it has been reported that COVID-19 patients show a prominent downregulation of ACE2 expression in the vascular endothelium, accompanied by immune infiltration and myocardial fibrosis (Bois et al., 2021; Wang et al., 2022a).

Furthermore, it has been suggested that because of an impaired ACE2, dysregulation in the balance of the levels of its substrates and products in both plasma and tissues may occur (Osman et al., 2021; Zhang et al., 2021; Wang et al., 2022). For instance, due to the decreased ACE2 hydrolytic activity, Ang II increases over Ang-(1-7), therefore overstimulating the AT1R and over activating the classical axis of RAS, which can eventually translate into hypertension, thrombosis, lung edema, fibrosis, and hyper−inflammatory reactions (Pucci et al., 2021; Zhang et al., 2021). The AT1R overactivation also exacerbates inflammatory response by the microglia conducing to the release of glutamate from nerve cells, increasing the production of ROS and NOS, which produce BBB permeability and the consequent neutrophil and T cell infiltration to the ischemic region leading to sepsis and neurotoxicity (Kaushik et al., 2020).

Ang II has hydrophilic features; therefore, it does not cross the BBB. Although, elevated circulating levels of Ang II in certain pathologic conditions, such as hypertension, provoke disruption of the BBB integrity, allowing access to circulating Ang II (Katsi et al., 2020). Evidence “suggests that peripheral and local RAS, *via* disruption of the BBB may promote exacerbated sympatho-excitatory activity and neurogenic hypertension” (Katsi et al., 2020). Another molecule crucially involved in the RAS is aldosterone. Besides being produced in the adrenal gland, evidence suggests that extra-adrenal tissues may synthesize aldosterone as it occurs with the brain. Brain tissue from mice exposed to a high-sodium diet shows an increased *CYP11B2* expression, indicating that sodium could regulate the expression of aldosterone in this organ (Geerling and Loewy, 2009; Gomez-Sanchez et al., 2010). Currently, few studies have demonstrated the relationship between aldosterone and the severity of COVID-19; it is known that high levels of aldosterone in elderly patients could increase the risk of mortality from cardiovascular complications due to its proinflammatory and atherosclerotic functions (Campana et al., 2022).

Human brain pericytes, critical components of the BBB, have been shown to express both AT1R and AT2R, with a predominant expression of AT1R (Noureddine et al., 2020). Both receptors mediate the response of pericytes to Ang II, characterized by increased production of Nox4, accompanied by an elevation of ROS. ROS increase BBB permeability by impeding the expression or function of the tight junction proteins; it also contributes to adverse vascular remodeling and vessel rarefaction that disturbs blood flow (Noureddine et al., 2020). Furthermore, Ang II might trigger pericyte loss and promote BBB impairment (Noureddine et al., 2020). The BBB leakiness leads to the accumulation of serum proteins, oligodendrocyte progenitor cell (OPC) activation, endothelial transcytosis, microglial activation, and aberrant TGF-β/Smad2 signaling activation, which together provoke brain damage (Sun et al., 2021). Also, BBB damage could make it more permeable to SARS-CoV-2 SNC invasion.

Moreover, Ang II is considered one of the primary atherosclerotic mediators. It regulates the expression of adhesion molecules (e.g., VCAM-1, ICAM-1, P-selectin), cytokines, chemokines, and the secretion of growth factors within the arterial wall (Montecucco et al., 2009). On the other hand, RAS can regulate the activation of the complement system in both atherosclerosis and renal injury (Montecucco et al., 2009). Hosomi et al. (2005) demonstrated that intravenous infusion of Ang II increases cerebral edema and mortality after cerebral ischemia-reperfusion injury (Hosomi et al., 2005). These results suggest that the accumulation of Ang II leads to endothelial dysfunction, which could produce severe cerebrovascular manifestations such as intracerebral hemorrhage (ICH), ischemia stroke (IS), and hemorrhagic stroke (HS) (it is calculated that the combined prevalence of cerebrovascular diseases in COVID-19 patients is ranging between 0.5 and 5%) (Tsivgoulis et al., 2020).

Another group of peptides that ACE2 metabolizes is apelins’. It has been reported that parental apelins induce the internalization of the apelin receptor (APLNR), which is mediated by β−arrestin signaling (Zhang et al., 2021). On the other hand, metabolized apelins signal toward the Gi pathway of the APLNR rather than the β−arrestin−induced internalization pathway. There is increasing evidence that ACE2 down−regulation derived from COVID−19 causes higher concentrations of parental apelin peptides and lower levels of metabolized apelins, thus interfering with “the beneficial anti−senescence, antithrombotic and angiogenesis homeostasis effects of the apelinergic system” due to the internalization of the APLNR (Zhang et al., 2021). Instead, the dysregulation of systemic apelins levels favoring parental apelins may cause some effects in the SNC, such as loss of structure or function of neurons, pro-inflammatory responses, oxidative stress, Ca^2+^ signaling, apoptosis, and autophagy (Luo et al., 2020).

Likewise, the peripheral accumulation of des−Arg^9^−bradykinin after ACE2 down-regulation in COVID-19 may provoke “neutrophil infiltration and inflammation, and increased fluid permeability into tissues causing edema” (Seltzer, 2020; Zhang et al., 2021). Also, kinetensin may exacerbate SARS-CoV-2 infection, inducing mast cell degranulation characterized by the release of histamine and other inflammatory mediators that increase vascular permeability, causing edema. Recently, an *in vitro* study of the BBB carried out in “human-induced pluripotent stem cell (iPSC)-derived brain microvascular endothelial cells” revealed that the BBB permeability is increased in response to neurotensin and bradykinin (Karamyan, 2021). Hence, in the COVID-19 context, these two peptides increase, making BBB prone to damage.

Once again, concerning COVID-19-associated neurological symptoms, it has been widely reported that females show a higher number of long-term persistent physical, cognitive, neurological, and neuropsychiatric symptoms than males (Jensen et al., 2022). Besides the underlying mechanisms that explain these sex disparities, authors have proposed that they rely on immune and RAS mechanisms, as well as age, as mentioned in previous sections. However, the biological and molecular explanations should be studied in more depth.

As shown in this section, various studies suggest that SARS-CoV-2 infection causes deregulation of the systemic RAS that favors the classical axis of this system. Likewise, evidence has been presented that this type of imbalance in RAS could affect the integrity of the BBB and cause oxidative stress, inflammation, apoptosis, and autophagy, which cause brain damage that can have acute clinical manifestations. If the balance in the RAS is not restored, this could develop neurodegenerative diseases. Therefore, regulating RAS might be a new therapeutic target to protect SNC from neural diseases. However, it is essential to note that these are still hypotheses as most of the available results derive from measurements in plasma and are only stratified by sex; thus, more studies are needed to determine the molecular mechanism in a tissue-specific manner, in this case in the brain.

## Brain renin-angiotensin system and neurological symptoms associated with COVID-19

The infection by SARS-CoV-2 has been deeply associated with neurological symptoms, possibly due to brain RAS imbalance (Harapan and Yoo, 2021).

As mentioned before, SARS-CoV-2 infection causes dysfunction of alternative RAS, contributing to the accumulation of Ang II and favoring the classical signaling pathway regulated by Ang II/AT1R. The lack of Ang II processing by ACE2 favors the binding of this peptide to AT1R, which can cause a local pro-inflammatory state that activates neuronal programmed cell death and the consequent dopaminergic neuronal loss, which could have some consequences as cognitive problems in patients recovered from COVID-19 as is shown in Figure 2 (Khezri and Ghasemnejad-Berenji, 2021). Besides the increment of inflammation, the upregulation of Ang II could elevate the oxidative stress levels leading to an impairment in the brain endothelial function, which could produce difficulties in learning and memory (Quarleri and Delpino, 2022). Classical studies in animal models show that the microinjection of losartan (an AT1R inhibitor) and Ang II unilaterally and bilaterally in the CA1 hippocampal area improves memory and learning in shuttle-box and step through behavioral test compared to control animals (Tashev and Stefanova, 2015). Though controversial, authors say that these observations may be explained by the fact that Ang II can bind both AT1R and AT2R; therefore, the blockade of AT1R would enhance the binding of Ang II to AT2R and, consequently, its activation, leading to the beneficial effects.

Although controversial, several studies show that SARS-CoV-2 can infect brain endothelial cells inducing vascular endothelialitis. Once the virus infects the endothelial cells of large cerebral arteries, it may induce a pathological activation of ADAM17. This may potentiate inflammation and diminish ACE2-mediated tissue protection through proteolytic shedding, preventing the Ang II processing within the brain tissue (Wang et al., 2022). Hence, Ang II triggers a thromboinflammatory process by incrementing ROS, pro-inflammatory cytokines expression, and vasoconstriction by the decrease in blood vessel diameter. This vasoconstriction reduces cerebral microcirculation, which could decrease cerebral irrigation, conducing to brain damage (El-Arif et al., 2021). A low expression of AT1R characterizes brain areas unaffected by the viral infection. Contrary, in these areas, AT2R is upregulated; thereby, Ang II can bind to this receptor with high affinity, triggering protective mechanisms such as neuronal survival (Figure 2; Fajloun et al., 2022).

Less urgent but more prevalent neurological manifestations of COVID-19 are anosmia and dysgeusia; the calculated prevalence for smell and taste disturbances varies according to the study ranging from 38.5–85.6% and 35.8–88.0%, respectively (Harapan and Yoo, 2021). Studies using human embryonic stem cell (hESC)-derived peripheral neurons show that these disturbances may be due to the direct infection of SARS-CoV-2 to peripheral sensory neurons, which were thought not to express ACE2; however, it has been determined that ACE2 expression is low but not zero in these cells. Furthermore, this study determined that the infection leads to the deregulation in the expression of genes associated with chemosensory functions, such as the olfactory neuronal marker genes fibronectin leucine-rich transmembrane protein (FLRT3), debrin 1 (DBN1), Taste 2 receptor member 31 (TAS2R31), chemosensory regulatory factors Yin Yang 1 (YY1), cannabinoid receptor 1 (CNR1), among others as is shown in Figure 2 (Lyoo et al., 2022).

Headache is considered a non-specific symptom of SARS-CoV-2 infection; however, COVID-19 patients who had never suffered from recurrent headaches before experience persistent incapacitating frequent headaches due to the virus infection (Harapan and Yoo, 2021). The co-localization of Ang II in the rat neurons and the human dorsal root ganglia (DRG) with substance P and calcitonin gene-related peptide (CGRP) proposes the regulation of Ang II in nociception (Patil et al., 2010). However, the mechanism involved in headaches due to SARS-CoV-2 has still not been elucidated (Dos Anjos de Paula et al., 2021).

Finally, myalgia, arthralgia, and fatigue are common musculoskeletal symptoms in COVID-19 patients (Tuzun et al., 2021). The relationship between these neurological manifestations and the classical RAS axis can be given by the induction of wasting skeletal fiber through the increment in protein degradation, the triggering of apoptosis, and the decrement of protein synthesis regulated by Ang II. Moreover, it has been demonstrated that Ang II inhibits satellite cell proliferation (muscle stem cells) and prevents skeletal muscle regeneration (Figure 2; Yoshida et al., 2013). An upregulation of Ang II has been proved in muscle pathologies such as sarcopenia and cachexia (Bahat, 2020).

## Renin-angiotensin system-mediated therapeutic approaches in COVID-19-associated neurological manifestations

According to the evidence above, the RAS is one of the main therapeutic targets in the battle against SARS-CoV-2 infection. Thus, studies encompassing genetic and pharmacological manipulation of RAS components suggest that targeting this system may be beneficial by slowing or reversing neurological dysfunctions secondary to COVID-19. As in any new disease, before we can develop new treatments and therapies, we must first deploy a “drug repurposing strategy” according to the available information. In that sense, several existing drugs target the different members of the RAS, modifying the balance mentioned above between the anti- and pro-inflammatory pathways controlled by the RAS. Of these drugs the ones that received particular attention in the initial months of the current pandemic were ACE inhibitors and ARBs (Arcos et al., 2020; Leclézio et al., 2020; Murray et al., 2021). ACE inhibitors commonly treat hypertension, heart failure, and myocardial infarction (Ooi and Ball, 2009). As their name implies, these drugs inhibit the conversion of Ang I to Ang II by ACE, reducing blood pressure, sodium retention, and cell growth (Messerli et al., 2018).

Moreover, Ang-(1-7) can be metabolized by ACE to Ang-(1-5). Therefore, the blockage of ACE would increase Ang-(1-7) levels. Also, shunting Ang I processing to Ang-(1-7) *via* neprilysin or thimet oligopeptidase would increase this peptide in the brain (Chappell, 2019).

On the other hand, ARBs directly antagonize Ang II at the AT1 receptors; these were developed as an alternative for patients unable to tolerate the adverse effects of ACE inhibitors, such as persistent cough (Momoniat et al., 2019). Since ACE inhibitors and ARBs increased the expression of ACE2, which acts as a receptor for SARS-CoV-2, there was increasing concern by some researchers that their use could lead to an increase in SARS-CoV-2-related symptoms and mortality (Fang et al., 2020; Patel and Verma, 2020). However, this does not seem to be the case, as new evidence showed that these do not affect the patient’s prognosis (Nouri-Vaskeh et al., 2021; Tleyjeh et al., 2022). Some researchers even proposed that the increase in ACE2 expression might be beneficial, thinking that one of the leading causes of severe symptoms is the downregulation of ACE2, caused by its internalization induced by SARS-COV-2 attachment to this receptor (Kow et al., 2020). As mentioned above, this process modifies the delicate equilibrium of the RAS, promoting vasoconstriction and pro-inflammatory response by Ang II. The increased concentration of ACE2 by ACE inhibitors and ARBs might return the RAS to balance, reducing the damaging inflammatory response (Tleyjeh et al., 2022).

Furthermore, ARBs have shown neuroprotective effects in different *in vivo* disease models, including that traumatic brain injury, stroke, dementia, and AD (Noureddine et al., 2020). However, it is essential to take into account that BBB-crossing ARBs (e.g., telmisartan, candesartan) or ACE inhibitors (e.g., captopril, lisinopril, fosinopril, perindopril, ramipril, trandolapril) (Glodzik and Santisteban, 2021) may show better results in neurologic, cognitive and memory impairments caused by COVID-19. The meta-analysis of several clinical studies reveals that using ARBs in COVID-19 patients is associated with reducing mortality (Guo et al., 2020). Moreover, the treatment with ACE inhibitors and ARBs favored the reduction of C-reactive protein and procalcitonin levels in patients with COVID-19 and hypertension (Cheng et al., 2020). For these reasons, ARBs and ACE inhibitors are currently approved as alternative treatments against SARS-CoV-2 infection (Kulkarni et al., 2022).

Another drug that affects the RAS is the renin inhibitor aliskiren. It inhibits the ability of renin to form angiotensin I by blocking the RAS at its origin (Wal et al., 2011). Being rarely prescribed, there is not enough information about the possible therapeutic role of this drug. However, molecular docking analysis suggests that aliskiren can block SARS-CoV-2 main protease M^pro^, which is essential for viral replication (Aly, 2020), although further clinical evaluation is necessary. Likewise, it has been reported that aliskiren upregulates cell viability and reduces inflammatory damage and apoptosis induced by Aβ accumulation in *in vivo* AD models (Chen et al., 2012; Lu et al., 2021).

Another strategy is enhancing the Ang-(1-7)/MasR pathway instead of the Ang II/AT1R pathway. Firstly, Ang (1–7) increases oxygen delivery to the brain through the cerebral microvessels by stimulating angiogenesis and enhancing blood flow *via* the upregulation of NO production (Noureddine et al., 2020). In a study by Xiao et al., it was observed that Ang-(1-7) suppressed Ang II-induced pro-apoptotic activity, ROS overproduction, and NO reduction in human brain microvascular endothelial cells (Xiao et al., 2015). In another study, infusion with Ang (1–7) promoted brain angiogenesis, enhanced cerebral blood flow, and reduced infarct volume and neurological deficits after permanent middle cerebral artery occlusion in rats (Jiang et al., 2014). However, the results of these *in vitro* studies should be taken with caution and tested in clinical studies to prove their safety and effectiveness.

Another interesting therapeutical approach is directly increasing the soluble form of ACE2 (sACE2), which promotes the expression of Ang-(1-7). There seems to be a correlation between sACE2 and the probability of recovering from SARS-CoV-2 infection (El-Shennawy et al., 2022); it has been demonstrated that the transfection of exosomes of endothelial progenitor cells with lentivirus containing human ACE2 cDNA (ACE2-EPC-EXs) has a protective effect on hypoxia/reoxygenation (H/R)-induced injury in cultured aging brain endothelial cells. Also, it was observed in a mouse DOCA-salt hypertension model that the overexpression of ACE2 in the brain “reduces oxidative stress and COX-mediated neuroinflammation, improves anti-oxidant and nitric oxide signaling, and thereby attenuates the development of neurogenic hypertension” (Sriramula et al., 2015).

Concerning the delivery mechanism, in recent years, there has been an increased interest in using nanoparticles or nanomaterials to deliver drugs in a more controlled way (Zhou et al., 2021). The use of nanoparticles is complex but has numerous advantages over the traditional methods, including but not limited to high stability, incorporation of both hydrophilic and hydrophobic substances alike, variable routes of administration (Patil et al., 2020), and control of the local RAS at the brain, the capacity to travel across the BBB by different methods (depends on the nature of the nanoparticle) (Zhang et al., 2016; Song et al., 2021). Although some research groups have developed ACE2-enriched nanoparticles that “trap” the SARS-CoV-2, inhibiting the viral attachment to target cells (Cai et al., 2020; Mesias et al., 2021), nanoparticles can be used to deliver any drug or peptide that modifies the RAS in favor of the patient. Lastly, we believe there is a molecule type with untapped potential, not only as a therapeutic target but also as a treatment against the SARS-CoV-2 infection: the micro-ARNS (miRNAs). MiRNAs are small (averaging 22 nucleotides) non-coding RNAs that regulate gene expression post-transcriptionally. They can recognize specific messenger RNAs and regulate their repression and degradation (O’Brien et al., 2018). Some miRNAs are upregulated in patients’ severe symptoms of SARS-CoV-2, and inhibiting these miRNAs could help the patient’s prognosis (Ying et al., 2021). Likewise, miRNAs targeting specific pro-inflammatory peptides (like cytokines and chemokines) could be used as a therapy to regulate the exacerbated inflammatory response presented in some patients (Naser et al., 2022). In theory, they could target any member of the RAS system with precision, depending on the needs of the patients. Moreover, miRNAs are small and stable enough to be used inside simple nanocarriers, like liposomes and micelles, with specific receptors or transporters to cross the BBB. This is fundamental if the objective is the local RAS in the brain or spinal cord (Poustforoosh et al., 2022).

Inhibition of the RAS pathway has brought to light the concern that ACE inhibition could increase ACE2 as observed in animal models, enhancing SARS-CoV-2 infection, which could lead to a form of the most severe disease. However, these findings have not been confirmed in humans. However, the serum increase in ACE2 could be beneficial since the virus could bind to this soluble ACE2, and its entry into host cells could be inhibited, thus avoiding its replication and dissemination. In addition, an increase in ACE2 could favor the processing of Ang II to other cytoprotective peptides. Hundreds of clinical studies have been conducted to determine the safety and efficacy of these inhibitors, primarily in patients with hypertension, diabetes, renal and cardiovascular disease. Recent meta-analyses conclude that both the use of ACE inhibitors and ARBs are safe and do not increase the risk of SARS-CoV-2 infection, nor does it increase the severity of COVID-19 (need for intensive therapy, use of mechanical ventilation, and death) (Lee et al., 2022). However, more studies are needed to determine the tissue-specific effect of RAS inhibitors, moreover, the use of micro-RNA needs deep investigations since important molecules of the RAS present single nucleotide variants (SNVs), which could modify the intervention by microRNAs. The use of RAS inhibitors, although it seems to be safe, should be taken with reservation because RAS modifications could enhance changes in other signaling pathways such as the kallikrei-bradykinin system, causing probable adverse effects (Schieffer and Schieffer, 2022).

## Conclusion

SARS-CoV-2 was initially identified as a respiratory virus, although it is now known to affect the whole body, even the nervous system. COVID-19-associated neurological symptoms include loss of taste and smell, headaches, stroke, delirium, and brain inflammation. SARS-CoV-2 can directly infect the CNS; however, the neurological effects may be caused by immune activation, neuroinflammation, and damage to brain blood vessels, which could probably be a consequence of the dysregulation of both local and brain RAS, as a result of the binding of SARS-CoV-2 to ACE2.

The disruption of ACE2 and the consequent accumulation of Ang II and other peptides (kinetensin, parental apelins, des−Arg^9^−bradykinin, and neurotensin), and the overactivation of AT1R, could lead to vasoconstriction dysregulation in blood pressure, pro-inflammatory and pro-oxidant states conducing to brain damage. Therefore, the brain ACE2 expression levels and the pattern of this expression might be important for the individual risk, potential symptoms, and treatment outcomes of the neurological manifestations associated with SARS-CoV-2 infection.

In addition, important sex-related differences in the neurological COVID-19-associated symptoms have been reported. In that sense, women seem to be more prone to develop physical, cognitive, neurological, and neuropsychiatric symptoms compared to males. This phenomenon could be closely related to the RAS profile disparities between males and females. However, this is an open field to explore in more depth.

**FIGURE 3:**
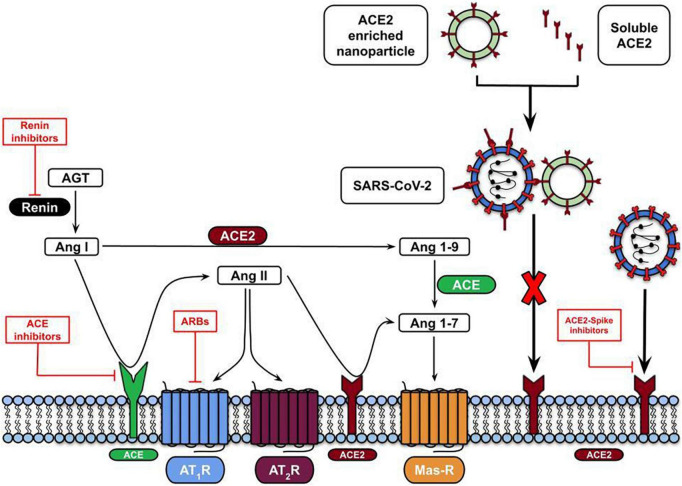
RAS-mediated therapeutic approaches against SARS-CoV-2 infection. The therapeutic approaches involve using ARBs (Angiotensin II receptor blockers) to inhibit the binding of Ang II to AT1R (red lines) or virus entry blocking by using the ACE2 soluble form or embedded in nanoparticles.

As discussed in this review, controlling the balance of systemic and brain RAS is promising to prevent or treat short and long-term neurological COVID-19-associated symptoms (Figure 3). However, the safety and benefits of using RAS inhibitors are still under debate, so more studies are needed and their use should be treated with caution.

## Author contributions

H-SV and LA-MG: writing—original draft. H-SV, LA-MG, AM-U, RV-S, and JA-G: writing—review, validation, and formal analysis. JC-R and GE: revising and supervising. All authors contributed to the article and approved the submitted version.
